# ‘Walk this way’: results from a pilot randomised controlled trial of a health coaching intervention to reduce sedentary behaviour and increase physical activity in people with serious mental illness

**DOI:** 10.1186/s12888-019-2274-5

**Published:** 2019-09-18

**Authors:** Julie Williams, Brendon Stubbs, Sol Richardson, Cathy Flower, Lucy Barr-Hamilton, Barbara Grey, Kathryn Hubbard, Gilda Spaducci, Fiona Gaughran, Tom Craig

**Affiliations:** 1 Health Service and Population Research Department, Institute of Psychiatry, Psychology and Neuroscience, King’s College London, London, UK; 2 Physiotherapy Department, South London and Maudsley NHS Foundation Trust, London, UK; 3 Psychosis Studies, Institute of Psychiatry, Psychology and Neuroscience, King’s College London, London, UK; 4 Addiction Department, Institute of Psychiatry, Psychology and Neuroscience, King’s College London, London, UK; 5 UK Centre for Tobacco and Alcohol Studies, Nottingham, UK; 6 Psychosis Clinical Academic Group, South London and Maudsley NHS Foundation Trust, London, UK; 7 National Psychosis Service, South London and Maudsley NHS Foundation Trust, London, UK

**Keywords:** Sedentary behaviour, Physical activity, Serious mental illness, Psychosis, Metabolic syndrome

## Abstract

**Background:**

Cardiovascular disease (CVD) is the leading cause of premature death among people with serious mental illness (SMI). Sedentary behaviour (SB) is an independent risk factor for CVD and mortality and people with SMI are highly sedentary. We developed a health coaching intervention called ‘Walk this Way’ to reduce SB and increase physical activity (PA) in people with SMI and conducted a pilot randomised controlled trial (RCT) to test its feasibility and acceptability.

**Methods:**

We randomised people with SMI from three community mental health teams into either the WTW intervention or treatment as usual. The WTW intervention lasted 17 weeks and included an initial education session, fortnightly coaching, provision of pedometers and access to a weekly walking group. Objective SB and PA were measured with accelerometers. Cardiometabolic risk factors and wellbeing measures were collected.

**Results:**

We recruited 40 people of whom 33 (82.5%) were followed up. 13/20 (65%) of participants allocated to the coaching intervention completed it. In the intervention group SB decreased by 56 min and total PA increased by 32 min per day on average which was sustained 6 months later. There was no change in PA or SB in the control group. When interviewed, participants in the intervention found the intervention helpful and acceptable. No adverse events were reported from the intervention.

**Conclusions:**

The intervention was feasible and acceptable to participants. Preliminary results were encouraging with improvement seen in both SB and PA. A larger study is needed to assess the effectiveness of the intervention and address any implementation challenges.

**Trial registration:**

ISRCTN Registry identifier: ISRCTN37724980, retrospectively registered 25 September 2015.

## Background

People with serious mental illness (SMI) such as schizophrenia, bipolar disorder and major depression die up to 20 years earlier than the general population; largely due to physical health conditions [1–3]. Recent global meta-analyses have demonstrated that people with SMI are at greatly increased risk of metabolic syndrome (MetS) [4], diabetes [5], and cardiovascular disease [6]. The cardiometabolic health of people with SMI in England is notably poor with a recent study demonstrating that 57% (*n* = 175) met the criteria for MetS while 20% had diabetes [7].

Addressing this disparity will require a multifaceted approach. Addressing lifestyle and specifically lack of physical activity (PA) has been identified as a key objective [8]. There is robust evidence in the general population that physical activity is broadly as effective as pharmacological interventions at preventing cardiovascular disease and associated mortality [9]. In addition to the protective effects of physical activity, there is now strong evidence that higher levels of sedentary behaviour (SB) (any waking behaviour characterized by an energy expenditure ≤1.5 metabolic equivalents (METs), while in a sitting, reclining or lying posture) [10] is independently associated with diabetes, cardiovascular disease and associated mortality [11].

Previous work has shown that people with SMI engage in very high levels of SB (12.6 h per day based on objective measures), equating to approximately 3 h per day more than the general population [12], and engage in low levels of PA [13]. A number of barriers contribute to this, including low mood and lack of support to increase PA [14]. Despite the high levels of SB and low levels of PA, very few interventions have specifically set out to address this and a recent systematic review [15] identified that no study had previously attempted to reduce objective SB in people with SMI. Given this evidence, we developed a health coaching intervention to reduce SB and increase PA in people with SMI living in the community called ‘Walk this Way’ (WTW).

In this paper we report the results of a pilot study of the intervention. This was done as we are aware that large expensive RCTs with this population have failed due, in part, to not being able to recruit participants [16] or not being feasible to implement in routine clinical practice [17, 18], and it is therefore essential to identify which of these barriers may be most relevant for WTW before conducting a fully powered randomised controlled trial. Eldridge and colleagues [19] recommend carrying out randomised pilot studies where ‘ the future RCT, or parts of it, including the randomisation of participants, is conducted on a smaller scale (piloted) to see if it can be done’ (p 14/15). This also provides clues as to the potential benefit of the intervention and a broad guide to the likely sample size required for a fully powered clinical trial.

## Methods

### Aims

The primary aim of this pilot RCT study was to establish the feasibility and acceptability of the WTW intervention by evaluating if participants diagnosed with serious mental illness (SMI) could be recruited and accept randomisation to the intervention, whether they would attend the recommended number of coaching sessions, attend an optional walking group and be willing to wear an accelerometer and provide blood samples for the intended primary and secondary outcomes that we would wish to use in a later powered trial.

Secondary aims were to estimate whether WTW had the potential to reduce SB and increase PA. In addition, we collected data on the impact of WTW on physical health measures related to cardiometabolic risk such as glucose regulations, blood pressure, waist circumference and other indicators of MetS.

### Methods/design

The methods and design of the WTW study have been published elsewhere [20]. Briefly, the RCT was undertaken in a Community Mental Health Team in South London between September 2015 and October 2017. We undertook a small process evaluation as part of the study in which we interviewed intervention participants to find out their views of the intervention. All participants gave informed consent and research ethics approval was obtained from the City Road and Hammersmith NRES Committee (15/LO/1188). The trial was registered in the ISRTCN Registry (ISRCTN37724980).

### Participants and sample size

Eligibility criteria were: a diagnosis of any SMI (ICD-10 clinical diagnosis of a schizophrenia spectrum disorder (F20–29), bipolar affective disorder (F31) or serious depression (F32.3); meeting any one of the following criteria as determined by a care coordinator (case manager): i) overweight, ii) at risk of or have diabetes, iii) in the clinician’s view, have a sedentary lifestyle, iv) or smoke tobacco; ability to provide informed consent; ability to understand English and over 18 years of age.

Our exclusion criteria were: under the age of 18, not having a diagnosis of SMI and unable to give informed consent.

Based on recruiting an adequate sample to assess the feasibility of the study with the resources available [21], we aimed to recruit 40 participants assuming around 20 would be assigned to the intervention arm.

### Procedure

Care coordinators (case managers) were asked to identify and refer eligible service users. All service users referred to the study who met our criteria were sent a letter explaining the study with a follow-up telephone call a week later. Those who expressed an interest in participating met a researcher who explained the study and were given written information, and the opportunity to ask any questions. After obtaining informed consent, baseline measures outlined in “Secondary outcomes” section were completed and participants asked to wear an accelerometer for a minimum of four consecutive days. After completion of the baseline measures, participants were randomised to either intervention or control.

A follow-up assessment was undertaken following the end of the intervention (i.e. after 17 weeks) where all measures apart from the sociodemographics were repeated. At 6-month follow up accelerometer data, blood pressure, body mass index, and waist circumference data were collected. These assessments were done by research workers who were blind to allocation status. Participants were reimbursed £10 for completing measures and £10 for wearing the accelerometer at each data collection point.

### Randomisation

We used simple randomisation and the randomisation was done by a researcher independent of the study using the random sequence generator (https://www.random.org). The researchers conducting the baseline assessment were unaware of which arm the participant had been allocated to when completing the baseline assessment and took an unopened envelope with randomisation status to the baseline assessment. Participants were informed of their allocation when baseline measures were complete by the researcher opening the envelope with them.

### Data collection

#### Primary outcome: the acceptability, feasibility and recruitment rates in the study

We measured:

(i) Time required (in months) to recruit 40 participants.
(ii) How many people needed to be approached to recruit 40 participants.
(iii) How many participants recruited into the study completed the intervention.
(iv) how many coaching sessions participants completed (out of the intended total of 8)
(v) how many participants attended the walking group at least once
(vi) satisfaction with the intervention
(vii) ability to collect all outcome data from all participants

#### Secondary outcomes

##### Average SB and PA time per day

All participants were asked to wear a wrist-worn GENEActiv accelerometer for at least 4 days at baseline and in the week prior to the end of treatment and the 6-month follow-up points. The accelerometer recorded how many minutes per day each participant was sedentary, and engaged in light, moderate and vigorous physical activities. Specifically, a recording is made of each 60 s period (called an ‘epoch’) which is classified by the accelerometer as either sedentary, light, moderate or vigorous PA. The cut-off points were defined according to Metabolic equivalents (METs) of sedentary (< 1.5 METs), light (1.5–3.99 METs), moderate (4.00–6.99 METs), and vigorous (> 7+ METs) based on standardised algorithms with high sensitivity and specificity [22].

We also collected information on other outcomes at the point of the baseline assessment and at each follow up:

1. Fasting lipids (total cholesterol, High Density Lipoprotein (HDL), Low Density Lipoprotein (LDL) and triglycerides, high sensitivity C reactive protein (CRP), insulin levels and blood glucose levels.
2. Blood pressure, waist circumference, weight and height.
3. Details of gender, age, ethnicity, living arrangements, smoking status, and self-reported psychiatric diagnosis.
4. Using relevant information from these assessments to determine the presence or not of a MetS ascribed according to the IDF definition and criteria of central obesity, raised triglycerides, reduced HDL cholesterol, raised blood pressure and raised fasting plasma glucose [23]

### Walk this way intervention and treatment as usual

The Walk this Way intervention follows the principles of the COM-B model of behaviour change [24] to address capability, opportunity and motivational barriers to reducing SB and increasing PA. We used an individually tailored coaching model that took account of capability limitations related to the participant’s general health and addressed opportunity and motivational barriers though a combination of didactic education, coaching and a walking group.

More specifically the WTW intervention consisted of the following components:

#### Initial education session

This session was adapted from the Walk, Address Sensations, Learn About Exercise, Cue Exercise Behavior for SSDs (WALC-S) program [25, 26] which is a motivational intervention based upon self-efficacy theory. We adapted the group sessions and introduced the concept of SB and the harms associated with it, along with strategies to sit less and move more including disrupting prolonged periods of sitting. The sessions also introduced the benefits of being more active and gave information, support and motivation to help participants to be less sedentary in their daily routines. All participants received a Yamax Digi-Walker CW-700 pedometer to self-monitor their daily activity levels and record this on an individualised calendar. The principles of coaching were introduced within the education session and the participants were introduced to their ‘coach’.

#### Health coaching

The health coaching component used the REACH© model of coaching. This model ‘emphasises accountability more explicitly than some other frameworks and models and involves connecting the head (thinking), heart (feeling) and hand (doing) to achieve self-identified goals’ [27]. Participants met with a coach for 30 min every 2 weeks. The participant and coach used these sessions to address any barriers to reducing SB and engaging in PA. The coaches had training in the REACH model and monthly supervision sessions with a coaching specialist throughout the intervention period.

#### A weekly walking group

All participants were invited to an optional weekly walking group led by the two coaches. This group met for approximately 2 h with the walks predominantly taking place in local parks. In addition to the benefits from exercise, there was an emphasis on the social aspect of group participation.

##### Control conditions

Participants in the control group received treatment as usual which consisted of care co-ordination plus written information on the benefits of increasing activity levels.

### Data management

All data from participants were anonymised. Data quality was enforced by having range checks and valid values. JW and BS entered the data.

We collected all instances of adverse events including injury, medical consultation or hospitalisation for either mental or physical problems, any mental health relapse or Crisis Team/ Emergency room contact.

The final dataset was accessed by the Principal Investigator and the research team only.

### Analysis

We calculated the percentage of people approached who participated in the intervention, how long it took us (in months) to recruit 40 people, how many participants completed the intervention and how many coaching sessions they attended, how many participants attended the walking group more than once, and satisfaction with the intervention. We also recorded the number of participants who refused to have any of the outcome data collected.

We tested whether secondary outcome measure scores (total minutes of SB per day and total minutes of light, moderate and vigorous, and total PA per day) differed between participants in the intervention and control arms at baseline. Next, we compared secondary outcome measure scores between participants in each trial arm at each follow-up (at end of intervention and 6 months later). We also investigated changes in pre and post test scores between baseline and each follow-up.

The analysis employed two-tailed Student’s t-tests for independent samples in each of the outcome measures between the control and intervention arms. While the use of q-q plots indicated that the relevant distributions of the outcome measures were near-normal for all tests, we tested for equality of variances using Levene’s robust statistic and the Shapiro-Wilk test was used to assess the normality of the distribution of the outcome measures. The tests confirmed equality of variances for the outcome measures for all test samples. In a minority of cases, however, the Shapiro-Wilk test found these distributions to deviate from normality. While the primary analysis used the t-tests, we also performed a sensitivity analysis by re-running the analysis using a non-parametric test (Mann-Whitney U test) without the assumption of normality.

We undertook semi-structured interviews with participants who took part in the intervention as part of a process evaluation to find out how participants experienced the intervention and if there are any parts of the intervention they would want changed. We used thematic analysis to identify the main themes.

## Results

### Primary aim

We attempted to contact all 205 people identified by care coordinators as eligible for the intervention by letter followed by a phone call. Of these, 100 could not be contacted at the address/telephone number provided. The researchers had contact with 105 using these methods, of whom 40 (42%) agreed to participate. The two main reasons for declining were being busy and having things to do (30 people) and being happy with their physical health and level of activity (15 people).

The demographic characteristics of the 40 participants are summarised in Table 1. The mean age of participants was 43 years (range: 20–56),55% (22/40) were male and 75% (30/40) were diagnosed with psychosis.

**Table 1:** Demographics of participants

	Category	Intervention (*n* = 20)	Control (*n* = 20)
Gender	Female	7	11
	Male	13	9
Ethnicity	White	6	5
	Black	12	8
	Asian	1	2
	Mixed	1	4
	Other	0	1
Live with	Alone	12	7
	With others	8	13
Diagnosis	Schizophrenia	12	10
	Bipolar	3	2
	Psychosis	2	1
	Other	3	7
Smoking status	Smoker	11	12
	Non-smoker	9	8

**Fig. 1:**
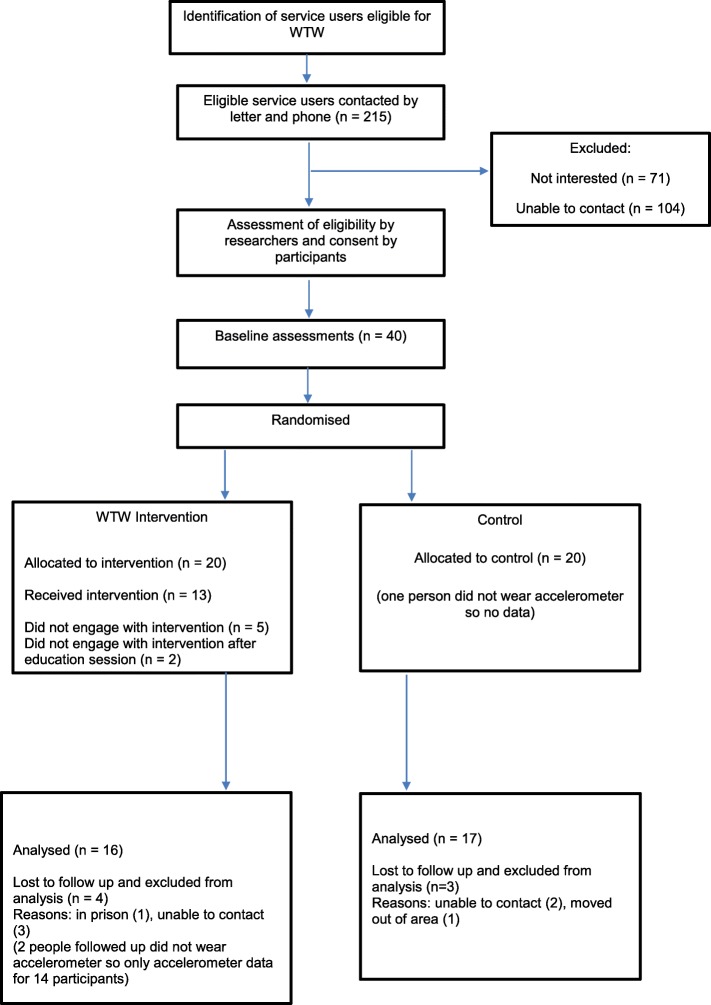
Walk this Way Flow diagram. Flow diagram of study recruitment

Follow-up data was available for 33 participants, resulting in a retention rate of 82.5%. Of the 20 people in the intervention arm, 15 attended the education session, 13 at least one coaching session and 8 joined the walking group (Fig. 1).

Eight participants attended all eight coaching sessions, one attended seven sessions, one attended six sessions, two attended four sessions and one attended one session. The participant who attended six sessions finished as they felt that they did not require any more sessions, one participant who attended four sessions was unable to attend more sessions as she had got a job, Of the 8 participants in the walking group some attended every week whilst others attended more sporadically. Where the reasons are known for this it appears that it was due to fluctuating mental health.

In the process evaluation we used semi-structured interviews with intervention participants to understand their experience of the intervention. Five participants agreed to be interviewed. Quotes from these interviews are in Table 2.

**Table 2:** Participant experience of the WTW intervention

Reason for taking part	‘I thought it would be a good idea in helping me to look at health and fitness, I was worried about my health’ (F/45yo)
	‘Well I got two main reasons. One was because I wanted to lose weight and another was because I wanted to be active and healthy’ (F/36yo)
	‘main reason to hopefully lose weight and get healthier’ (M/20yo)
Education session	‘I found out that walking helps you lose weight. Being more active, like being inactive causes a lot of diseases, so you need to be more active. Just even walking.’ (F/36yo)
	‘That any exercise is good, it doesn’t have to be you know some great big long like a run or something, just little things like walking to the shops and back.’ (F/45yo)
Coaching sessions	‘I felt it was good because it was about what I wanted to do and having support around that because it’s very easy to say I want to do this, and I want to do that but you need that sort of support to get yourself going so it sort of got me in the mind set to want to do some activities’ (F/45yo)
	‘The biggest positive was … meeting with (coach), setting up goals and having someone to, to erm check up to see if your achieving your goals and give you advice, yeah that was the biggest positive’ (F/32yo)
	‘The coaching helped me stay motivated, helped make myself more confident in the group.’ (M/20yo)
	‘when I’m not hitting my targets I know that I could be doing more. Sometimes, I might, instead of going to the shop local I might go the shop further’ (M/39yo)
Walking group	‘You get the chance to go out and meet people, go to different parks … You learn new things, you learn new places to go, things I want to do with my son.’ (F/36yo)
	‘Yes I still attend. Its very fun, get to meet people, talk to people, walk around in the parks- it’s very therapeutic.’ (M/20yo)
	‘I like meeting new people and I like going to new places’ (M/39yo)
Overall	‘It was good, just to be educated about walking and the benefits you can get from it and keeping yourself active.’ (F/36yo)
	‘it was really good because every day I wanted to, and also the weather was really good, I always wanted to go out, I didn’t want to be bored at home, you know, for some reason my sleeping improved as well, because I was more active and more tired’ (F/32yo)
	‘I try to use the advice. Walk mum up and down the road which wouldn’t have done before WTW- wouldn’t have done it before as be too drowsy but now I fight through it.’ (M/20yo)

Three participants refused to wear accelerometers (two at follow up only) and 3 refused blood tests. All other outcome data was collected.

### Secondary outcomes

#### SB and PA

Overall, only 24 participants had four full days accelerometer data at baseline. There was no significant difference between intervention and control groups in the number of days the accelerometer was worn (intervention group 4.2 [1.2] days, comparison 3.8 [1.2] days) nor was there any significant association between baseline SB and subsequent dropout from the study (completers SB baseline = 561.7 [83.9] vs dropout 565.7 [93.8]). Two intervention participants did not have accelerometer data at follow up so accelerometer data at follow up is for 31 participants. At 6 months 21 participants had accelerometer data, with 8 in the intervention group and 13 in the control group. We therefore calculated the mean minutes for SB and each classification of PA over three consecutive days for which we had the most complete data at baseline. Table 3 shows the mean (se) minutes of SB, light, moderate/vigorous, and total PA per day at each assessment point. Levels of activity did not differ significantly between the two groups at baseline. However moderate/ vigorous, and total PA were significantly higher in the intervention group than in the control group at the first follow-up while daily minutes of SB were significantly lower. Similar results were found at the 6-month follow-up with light, moderate/vigorous, and total PA significantly higher in the intervention group and minutes of SB significantly lower in the intervention group. Given the small sample size, no attempt was made to impute missing data so that when analysis was restricted to those participants with complete data at baseline and at each follow up point, only the change (reduction) in sedentary scores between baseline and both follow up points was statistically significant. The results of an analysis using the Mann-Whitney U test gave the same significant associations as found using t-tests (Table 4).

**Table 3:** Activity level - mean (se) minutes/day

Activity	Baseline *n* = 39			Follow-up *n* = 31			6-month follow up *n* = 21		
	Intervention	Control	*P*	Intervention	Control	*p*	Intervention	Control	*P*
A. Sedentary	577.2 (9.8)	549.2 (19.1)	0.324	520.9 (36.2)	637.9 (30.4)	0.019	508.2 (19.4)	661.2 (33.5)	0.003
B. Light	84.2 (7.5)	69.4 (4.4)	0.103	91.4 (6.5)	67.2 (12.8)	0.126	98.04 (10.2)	62.3 (6.6)	0.006
C. Moderate/ Vigorous	126.4 (15.2)	97.1 (10.9)	0.128	166.5 (22.9)	105.1 (146)	0.026	186.9 (20.0)	109.9 (23.4)	0.035
Total B + C	210.7 (20.8)	166.5 (13.9)	0.089	257.9 (27.0)	172.3 (21.9)	0.019	284.9 (27.9)	172.3 (29.3)	0.018

**Table 4:** Change in activity from baseline to follow-up and 6 months (mean [se] mins/day)

Activity	Baseline to Follow up *n* = 31			Baseline to 6 months *n* = 21		
	Intervention	Control	*P*	Intervention	Control	*p*
A. Sedentary	−56.1 (27.6)	87.6 (17.6)	< 0.001	− 64.9 (30.3)	96.5 (23.0)	<.001
B. Light	- 0.7 (8.6)	0.1 (12.4)	0.667	−8.3 (14.5)	−4.4 (7.7)	0.795
C. Moderate/ Vigorous	37.2. (15.4)	9.0 (9.5)	0.109	27.7 (24.4)	12.4 (13.0)	0.551
Total B + C	31.8 (21.3)	10.6 (12.7)	0.384	19.4 (33.0)	8.1 (19.3)	0.753

#### Physical health measures

While there was no statistically significant difference between the two groups on any of the biometric physical health measures at follow up, there were small changes in favour of the intervention group in waist circumference (intervention group from a mean of 111 [sd19.6] cm to 104 [sd16.5] cm, control 112 [sd13.4] cm to 110 [sd12.4] cm), and CRP (from 8.3 [sd9.8] mg/L to 2.7 [sd3.1] mg/L in the intervention group and from 6.4 [sd6.7] mg/L to 4.61 [sd4.4] mg/L in the control group). At baseline, 38 participants had sufficient data to determine the presence or not of the MetS. Of these, 23 (61%) met the criteria for MetS. At follow-up, of the 26 for whom we had data to calculate MetS, 15 (58%) met the criteria for MetS. three participants in the intervention group moved from MetS present to absent with none developing MetS over the course of the intervention. Two participants in the control similarly moved from MetS present to absent but 2 also moved from MetS absent to present.

## Discussion

### Primary outcome – feasibility, acceptability and uptake of intervention

Care coordinators had caseloads of between 15 and 30 and based on previous research in a similar cohort [7] we estimated that at least 60% of this caseload would be assessed as eligible. However, fewer people were referred to us than expected. Reasons given for this include ‘paternalism’-thinking clients too unwell to participate [28, 29], ‘gatekeeping’ [28], negative past experiences (both of care coordinators and service users) [29] and clinical staff feeling they did not have the necessary knowledge of research to feel comfortable discussing this with service users [30]. The latter may have been a significant contributor in this instance as there was a high staff turnover at the time of recruitment to the study, with new and locum staff perhaps less confident about applying the inclusion criteria, making risk judgements and suitability of patients for referral to the study [31].

Of those referred, we were unable to contact 105 people. All were approached by letter followed by a call to the phone number recorded in their case notes. Only two people responded to the letter. When contacted by phone, however, many people did not remember receiving the letter. Most phone numbers were mobile phone numbers and it may be that people were reluctant to answer calls from unknown numbers.

Of those who were approached and agreed to take part, drop-out was broadly in line with comparable studies of interventions for people with enduring SMI [32, 33] though more than others in specialised services [25, 34].

Only eight participants attended all three components of the intervention. The education session was attended by the most participants followed by take up of coaching and finally the walking group.

From the qualitative interviews we found that people enjoyed the intervention although the reasons they gave were quite variable and included understanding more about the importance of physical activity, being able to work on their goals and feeling supported with this, and for others the social aspect of the walking group were most important.

Before the pilot we did not know if participants would agree to wear the accelerometers for any length of time. They are not physically appealing or particularly comfortable and some participants had concerns about being remotely monitored. Only two out of 40 people refused to wear them after detailed explanation which is a helpful finding for future studies.

### Secondary outcomes – indicators of effectiveness

There was a clinically significant reduction in SB and increase in PA in the intervention compared to the control group which was maintained at 6 months post-intervention. However, it is also apparent that the intervention group were already somewhat more active at baseline and although this difference was not statistically significant we cannot entirely rule out the possibility that randomisation allocated a group of participants who were already more likely to be responsive to the intervention. Another important observation is that the accelerometer readings from both the intervention and control group indicated, using general population classifications, that participants were much more physically active than would have been expected from previous meta-analysis [13], even at baseline. The analysis was based on 3 days accelerometer use rather than four. As yet, there is little data concerning the optimum amount of data required to accurately measure SB. However a recent paper from a general population sample found 3.4 days to adequate to measure SB [35].

There were no statistically significant changes in any of the biometric indicators of physical health which is perhaps not unexpected given the small sample size and short follow-up period. The small reduction in CRP in favour of the intervention group is of interest as CRP is elevated in SMI [36, 37]. Moreover, reductions in CRP have been associated with reduced risk of cardiovascular mortality in the general population [38] and it plausibly reflects increased persistent physical activity as has been shown in other studies [39].

### Strengths and limitations

To the best of our knowledge this is the first RCT undertaken in England of an intervention to support people with SMI to be less sedentary and more physically active. Our main outcome assessment (activity measured by accelerometer) minimises inaccurate reporting and risks of biased assessment. Although the validity of accelerometers in objectively quantifying SB and PA has not been assessed in people with SMI, the accelerometer was able to detect a change in activity levels over time and between groups.

Our sample was ethnically diverse reflecting the local area. We were able to recruit to target, had 82.5% retention in the trial and the majority (65%) of participants completed the coaching intervention. Our preliminary results suggest that the intervention may be effective in reducing SB.

Recruitment took longer than expected and we encountered challenges with staff referral rates and in contacting potential participants. In subsequent trials we would consider different recruitment strategies, including employing researchers to screen for inclusion criteria rather than relying solely on care coordinators and taking a more proactive approach to advertising of the study.

### Clinical implications

If a larger study confirms the effectiveness of WTW, it will be necessary to consider how best to implement and deliver the intervention in busy teams with competing priorities, given the importance of supporting PA and reducing SB in this group. The implementation in this pilot was undertaken by two experienced Occupational Therapists in a research-friendly clinical environment with protected time and supportive colleagues. These are important aspects in ensuring the intervention was implemented as planned.

There may be alternatives to NHS teams and health professionals providing the intervention. Third sector and voluntary organisations already provide support for people with SMI and they may have capacity to take this on. Mainstream organisations with experience of running walking groups in the community may also be partners in running the intervention. Interest in the role of peer support workers in mental health is growing [40] and peer workers could be ideally suited to running this intervention.

### Research implications

This pilot work is encouraging, but a larger trial is now needed to demonstrate the clinical and cost-effectiveness of the intervention. Recruitment in the pilot was routed through care coordinators who were asked to assess a patient’s eligibility which may have created an unnecessary filter. Future work may need to address this issue, optimising service users’ awareness of research opportunities open to them and use of research registers such as the Trust ‘consent to contact’ register [38] where these exist. Once in the trial, retention rates were over 80% and completion rates for the intervention were good at 65% but further qualitative analysis of interviews from this and other studies by our group may also inform future strategies to optimise this.

A larger trial would also address the relative importance of the three main components and be powered to enable tests of mediators and moderators of treatment, perhaps formulated to map on to the capability, opportunity and motivational barriers described by the COM-B model of behaviour change.

As already mentioned, our participants in both arms were more physically active than reported previously published meta-analysis data [12, 13, 15]. One reason could be that the previous meta-analyses data is overwhelmingly based on cross sectional measurement, whilst our trial participants were recruited in a random manner to a trial that was investigating if it was possible to increase physical activity and reduce SB and were volunteers from an outpatient population with heterogenous diagnoses. Previous research in the general population has found that simply recruiting people to physical activity trials results in an increase in activity levels [41] this may have occurred in both groups in our feasibility study. Another consideration is that we utilised standardised general population cut points to measure physical activity and sedentary behaviour and this may have led to an overestimation of the amount of physical activity. Whilst this would have been constant over the course of the study and not have affected mean difference change, clearly future research is required to optimise objective monitoring of physical activity and sedentary behaviour in people with SMI and agree classification of accelerometer-measured PA into light, moderate or vigorous in that population.

## Conclusions

This is the first RCT of an intervention with the primary aim of reducing objectively-measured SB in people with SMI. Recruitment was to time and target and retention was 82.5%, suggesting the intervention was acceptable. There was a reduction in SB in the intervention group which was maintained after 6 months, suggesting the intervention may be effective. A larger effectiveness trial is warranted to evaluate this.

## Data Availability

The dataset used and analysed during the current study are available from the corresponding author on reasonable request.

## Abbreviations

CVD: Cardiovascular disease
METs: Metabolic equivalent
MetS: Metabolic syndrome
PA: Physical activity
RCT: Randomised controlled trial
SB: Sedentary behaviour
SMI: Serious mental illness
WTW: Walk this Way
